# A Textbook of Physiology

**Published:** 1907-04

**Authors:** 


**A Textbook of Physiology.** By **Robert Tigerstedt,** M.D., professor of physiology in the University of Helsingfors, Finland. Translated from the second German edition and edited by **John R. Murlin,** A.M., Ph.D., Assistant Professor of Physiology in the University and Bellevue Hospital Medical College, New York. With an introduction in English by **Professor Graham Lusk,** Ph.D., F.R.S. (Edin.). Octavo, pp. 751. New York: D. Appleton and Company. 1906. (Price, Cloth, $4.00).

It is seldom that one picks up a scientific work which, while imparting a sense of solidity, lacks that high-browed heaviness of tone which makes its reading a duty and not a pleasure. This translation of Tigerstedt's physiology is such an one and aside from its general excellence as a textbook, which is accepted as a matter of course, in view of the author's reputation and the preeminence of its translator, there are a number of features which are so different from the usual books on physiology that they demand attention and hold interest. The author approaches his subject in a broadminded manner; not with mincing step or haughty mien. He simply and logically lays the foundation for the study in a convincing manner. To the foundation he adds the physiology of the cell, and the structure acquires strength by the addition of the story of the chemical constituents of the body. Metabolism and nutrition come then, and here is new thought aplenty, chief of which is that all energy transformations of the body rest on the principles of the conservation of energy. This is something novel and as yet no physiologist has suggested it. This chapter in connection with that on the circulation, being within the strict lines of the author's especial fields of investigation, is rich in new and important material.

Every portion of the work is masterly and whether considered from the standpoint of the specialist or the general practitioner is as nearly without the pale of honest criticism as a scientific work can well be. The illustrations are excellent and number over 300, sixty-three of which are in colors. It is the most completely and artistically illustrated book on physiology ever published. Furthermore, it is quite within the bounds of reason to say that it is the broadest-minded and most satisfactory physiology in English; and that is about equivalent to including all the other languages.

